# The Added Value of Intraoperative Hypnosis during Spinal Cord Stimulation Lead Implantation under Awake Anesthesia in Patients Presenting with Refractory Chronic Pain

**DOI:** 10.3390/medicina58020220

**Published:** 2022-02-01

**Authors:** Chantal Wood, Gaëlle Martiné, Gaëlle Espagne-Dubreuilh, Karine Le Goff, Maarten Moens, Lisa Goudman, Sandrine Baron, Romain David, Nicolas Naïditch, Maxime Billot, Philippe Rigoard

**Affiliations:** 1 PRISMATICS Lab (Predictive Research in Spine/Neuromodulation Management and Thoracic Innovation/Cardiac Surgery), Poitiers University Hospital, 86021 Poitiers, France; sandrine.baron@chu-poitiers.fr (S.B.); romain-david@hotmail.fr (R.D.); nicolas.naiditch@gmail.com (N.N.); Maxime.BILLOT@chu-poitiers.fr (M.B.); 2 Department of Spine & Neuromodulation, Poitiers University Hospital, 86021 Poitiers, France; 3 Clinique François Chénieux, Polyclinique de Limoges, 87000 Limoges, France; gaellemartine@gmail.com (G.M.); karine.legoff@chu-limoges.fr (K.L.G.); 4 Pain Center, Limoges University Hospital, 87000 Limoges, France; gaelle.espagne-dubreuilh@chu-limoges.fr; 5 Department of Neurosurgery, Universitair Ziekenhuis Brussel, 1090 Brussels, Belgium; mtmoens@gmail.com (M.M.); lisa.goudman@gmail.com (L.G.); 6 STIMULUS Consortium (reSearch and TeachIng neuroModULation Uz bruSsel), Vrije Universiteit Brussel, 1050 Brussels, Belgium; 7 Physical and Rehabilitation Medicine Unit, Poitiers University Hospital, University of Poitiers, 86021 Poitiers, France; 8 Prime Institute UPR 3346, Centre National de la Recherche Scientifique (CNRS), Institut Supérieur de l’Aéronautique et de l’Espace-Ecole Nationale Supérieure de Mécanique et d’Aérotechnique Poitiers Futuroscope (ISEA-ENSMA), University of Poitiers, 86360 Poitiers, France

**Keywords:** persistent spinal pain syndrome (PSPS), failed back surgery syndrome (FBSS), neuromodulation, chronic pain, surgery, adjunct therapy, conscious sedation

## Abstract

To improve pain relief for refractory pain condition, spinal cord stimulation (SCS) needs to target the dedicated neuronal fibers within the dorsal columns. Intraoperative feedback from the patient can optimize lead placement but requires “awake surgery”, allowing interaction between patient and surgeon. This can produce negative effects like anxiety and stress. To better manage these aspects, we propose to combine intraoperative hypnosis with awake anesthesia. Seventy-four patients (35 females, 22–80 years) presenting with chronic refractory pain, were offered intraoperative hypnosis during awake SCS lead implantation. Interactive conversational hypnosis was used as well as interactive touch, which was enhanced during painful moments during the lead intraoperative programming. All patients participated actively during the intraoperative testing which helped to optimize the lead positioning. They kept an extremely positive memory of the surgery and of the hypnotic experience, despite some painful moments. Pain could be reduced in these patients by using interactions and touch, which works on Gate Control modulation. Positive memory was reinforced by congratulations to create self-confidence and to induce positive expectations, which could reinforce the Diffuse Noxious Inhibitory Controls at the spinal level. Cooperation was improved because the patient was actively participating and thus, much more alert when feedback was required. Combining intraoperative hypnosis with awake anesthesia appears helpful for SCS lead implantation. It enhances patient cooperation, allows optimization of lead positioning, and leads to better pain control, positive and resourceful memory.

## 1. Introduction

Management of some refractory chronic pain conditions (failed back surgery syndrome, persistent spinal pain syndrome, complex regional pain syndromes, neuropathic pain, etc.) can be improved by using epidural Spinal Cord Stimulation (SCS) [1,2,3]. Conventional SCS needs to target the dedicated neuronal fibers within the spinal cord dorsal columns in order to produce substantial pain relief [4]. Surgical or percutaneous lead placement consequently remains critical to improve patient outcome. Although inter-individual anatomical similarities exist, each patient has specific spinal cord anatomy, which means that optimal lead positioning varies from one patient to another and should benefit from direct intraoperative patient feedback whenever possible.

Awake neurosurgical procedures with conscious sedation have been described in the 1970s and are now commonly used for brain tumor resection, allowing true and reversible interaction between patient and surgeon [5]. This concept can be transposed to SCS implantation, whatever the implantation method, using a percutaneous or a surgical lead. In this case, in order to minimize the invasiveness of surgical intervention and decrease patient discomfort during awake spinal surgery, minimally invasive procedures have been developed, such as the Minimal Access Spinal Technology (MAST) spinal approach [6]. Although this method may reduce patient discomfort [7,8,9], some studies have reported that these types of surgery performed under awake anesthesia can have a dramatic psychological impact during and after the procedure: anxiety, stress, discomfort and even post-traumatic stress syndrome (PTSS) [10].

For 35 years, clinical hypnosis has been used in operating rooms to manage procedural pain, stress and anxiety [7,8,9,11]. In a recent systematic review and meta-analysis, Noergaard et al. [8] reported that intraoperative hypnosis had a beneficial clinical impact on pain medication (i.e., sedatives and analgesics), perioperative affects (stress, depression, anxiety), and recovery (e.g., return of muscular strength and fatigue). To improve patient experience during SCS implantation, the combined use of intraoperative hypnosis and awake anesthesia while optimizing SCS lead positioning appears to be of interest but requires further investigation.

## 2. Materials and Methods

### 2.1. Study Population

Seventy-four patients (35 females, 22–80 years) presenting with chronic refractory pain, were offered intraoperative hypnosis during awake SCS lead implantation between 2016 and 2020 at the University Hospital of Poitiers (Table 1). A Minimally Invasive Access Spine Technology (MAST) approach was used for 34 cases implanted with a surgical lead. Percutaneous leads were implanted in 35 cases [6].

### 2.2. Hypnotic Procedure

All patients were seen for 30–60 min by the hypnotherapist, a pain physician, before the procedure (between a month and a day pre-surgery), in order to strengthen the alliance and reinforce the required learning, and again just before surgery in the recovery room. Preliminary data collection was conducted, in order to analyze the patient’s interests (hobbies, pets, cooking, journeys) and sensorial preferences (vision, audition, taste, smell, kinesthesia).

Hypnosis was described as a moment of focalized attention. Explanations were given about the surgical procedure and the importance of patient cooperation during the lead intraoperative programming. An experiential method using a graphite pencil was used to highlight the importance of the interaction with the hypnotherapist. The graphite pencil was used as a stimulus, warning the patient of the prick. The experience was renewed when the patient was interacting with his fingers and those of the therapist, asking him, for example, to press with his second finger, then jointly with the third and the fifth fingers. As the stimuli were felt differently and experientially, he was told that the more he would be active and interacting physically and verbally, the less he would feel the surgical procedure. He was also told that he would have local anesthesia (by lidocaine), analgesics, and sedatives by remifentanil, adjusted according to the level of pain, so that he would be as comfortable as possible. Another hypnotic technique, glove anesthesia, was used to prepare the patient for the injection of local anesthetic. The therapist also explained that he would be close to the patient, touching his hand, and that the patient could report any discomfort by pressing the hypnotherapist’s hand.

In the operating room, once installed, the therapist began to gently rub one of the patient’s hands, asking him to protect that hand and transfer the protection to the area of surgery, and to reinforce this protection by each stimulation he was receiving (cleaning the area with different products and rubbing it with sterile gauzes). During the injection of local anesthetic, the therapist interacted with the fingers of the patient in order to help him focus on another part of his body and reduce the pain of the prick and injection of the product. During surgery, conversational hypnosis was used, asking the patient to give details of what he enjoyed doing (going for a walk, cooking, playing with his favorite animal), thereby interacting by asking questions. During the most painful moments, physical interaction was reinforced, to keep the patient focused on another part of his body. When the lead was positioned, the patient was asked to «return» to the operating room and participate in the intraoperative tests. Depending on the test results, the lead would be fixed or repositioned if necessary. Conversational hypnosis was used again, with the active participation of the patient, unless he/she preferred not to speak. In these cases, the hypnotherapist took over.

At the end of the surgery, all the operating room staff congratulated the patient (applauding him). This was done so that the patient would keep a positive memory of the operation, create anchorage with a special moment, and realize that he possessed precious resources (positive resources are very important for a chronic pain patient). This experience also provided a positive emotional memory, which is of prime importance if the surgery is to be repeated. All patients were seen the evening after surgery or on the following day to obtain feedback on their experience of the surgical procedure.

## 3. Results

All patients had a highly positive experience of hypnosis and of what had happened during the surgery, despite some painful moments. They reported that touching the hypnotherapist’s hand had increased their sense of confidence and safety. They would also advise other patients to use hypnosis and proceed in the same way.

All patients participated actively during the intraoperative testing, which helped to optimize the lead positioning. Surgical outcomes appear to be likewise improved: while an operation could last from 1 to 4.5 h, the patients did not feel that it took a long time. For two patients with pelvic pain, exact positioning of the lead was possible but took time. In one case, the test phase and lead positioning took up 2 out of the 3 h of surgery. For these two patients, previous surgery had been performed under general anesthesia by the surgical team referring these patients to our Hospital. Previous procedures attempting to target the appropriate fibers and generate adequate paresthesia in the painful territory had been unsuccessful.

## 4. Discussion

Using touch, developing conversational hypnosis, and strengthening resources by congratulating the patient at the end of the intervention are techniques used mainly with children. Touch is rare when interacting with adults. However, we are aware of the fact that A-alpha and A-beta stimulation will block transmission of the painful message transmitted by the A-delta and C fibers (Gate Control phenomenon).

Touch is important, as it reinforces the therapeutic relationship, generates trust and ensures presence, while showing the patient that he is not alone. It also activates and strengthens the patient’s resources, activates proprioception, regulates the autonomic nervous system, and generates grounding and security. Playing with the patient’s fingers and physical interaction is more of a pediatric/geriatric approach [12,13,14,15], allowing patients to be focused on something else during potentially painful gestures.

Our patients were more alert and participated more effectively in the intraoperative testing used to improve the exact positioning of the lead, even when duration of the surgery was long. This could not have been done without the support of hypnosis, helping the patient to regain energy and to use it as a means of comfortably continuing with the tests. Congratulating the patient is also inspired by pediatric techniques, and congratulating a chronic pain patient is of particular importance insofar as he is very often considered as a failure, whom no one wishes to encounter. Congratulated by the entire operating room staff, the patient realizes that he can be respected, that he can do something extraordinary and that he possesses precious resources within himself. This builds self-confidence that can be used at any time of his or her life. Another objective was to provide a positive memory. The more fun the patient has during surgery, the more he talks about what he loves, the more he retains an emotionally positive memory, and the greater his expectations for the future. If, on the other hand, his expectations are negative, conditioned pain modulation (CPM) is inhibited [16], leading to more pain [17] if surgery has to be repeated.

Hypnosis appears to be really helpful in SCS lead implantation. It enhances the cooperation of the patient, leading to better positioning of the lead. Interacting with the patient during surgery, asking him to be active and to focus on something else, creating a positive memory and congratulating him at the end of the procedure appear to be key factors in success, contributing to a positive and resourceful memory, which can be used by the chronic pain patient whenever the need arises, particularly in any future surgery.

## 5. Conclusions

Hypnosis appears to be genuinely helpful in SCS lead implantation. It enhances the cooperation of the patient, leading to better positioning of the lead. Interacting with the patient during surgery, asking him to be active and to focus on something else, creating a positive memory and congratulating him at the end of the procedure appear to be key factors in success.

## Figures and Tables

**Table 1:** Characteristics of patients.

Variables	n/Mean ± SD
Age (years)	53.5 ± 12.5 (range: 22–80)
Sex	35 females/39 males
Pathologies	
PSPS-T2	49
Neuropathic pain	20
CRPS	3
Cluster Headache	1
Alcock syndrome canal	1
Pain predominance	
Radicular	53
Low back pain	11
Pelvic floor	5
Occipito-cervical	2
Cervical	1
Cluster Headache	1
High back pain	1
Implanted lead	
Percutaneous	35
Surgical	35
Sub-cutaneous	3
Percutaneous + sub-cutaneous	1

CRPS: Complex Regional Pain Syndrome; PSPS-T2: Persistent Spinal Pain Syndrome after surgery.

## Data Availability

Not applicable.
